# Burnout among Probation Officers in Poland and the Role of Preferred Styles of Coping with Stress

**DOI:** 10.3390/ijerph18010355

**Published:** 2021-01-05

**Authors:** Łukasz Wirkus, Anna Babicka-Wirkus, Robert Opora, Krzysztof Stasiak

**Affiliations:** 1 Institute of Pedagogy, Faculty of Social Science, University of Gdańsk, Bażyńskiego 4 St., 80-309 Gdańsk, Poland; lukasz.wirkus@ug.edu.pl (Ł.W.); robert.opora@ug.edu.pl (R.O.); 2 Institute of Pedagogy, Pomeranian University in Słupsk, Arciszewskiego 22A St., 76-200 Słupsk, Poland; 3 Department of Material Criminal Law and Criminology, Faculty of Law and Administration, University of Gdańsk, Bażyńskiego 6 St., 80-309 Gdańsk, Poland; krzysztof.stasiak@prawo.ug.edu.pl

**Keywords:** probation officers, burnout, coping with the stress, job stress, justice system in Poland

## Abstract

The current article examined the relationship between preferred styles of coping with stress and occupational burnout among probation officers in Poland. The probation system in Poland is unique in comparison to similar organizations in Europe and the world. It is characterized by two separate specializations in the area of performed tasks: probation officers for adults and for family and juvenile clients. The main purpose of the study was to assess the relationship between occupational burnout levels among probation officers (*n* = 390) and their preferred styles of coping with stress. Two psychological tools were used in the study: the Maslach Burnout Inventory (MBI) and the Coping Inventory for Stressful Situations (CISS). A linear regression analysis was carried out to explain the variance in occupational burnout. Occupational burnout was the dependent variable and the CISS scales were the predictors. In order to test the moderating role of the sociodemographic factors of gender, work experience, age, and probation specialization in the relationship between coping styles and occupational burnout, a range of moderation analyses using Hayes’ PROCESS macro on SPSS was carried out.

## 1. Introduction

Crime is among the most significant social problems. Efforts to combat and prevent crime are elements of every country’s social and criminal policy [1,2]. It is realized through the activities of various services, such as the courts, prosecution, prison service, police, social workers, and probation officers. The current study focused on the latter service, which significantly differs from the others due to the nature of its tasks. The work of probation officers is characterized frequent and direct contacts with clients as well as the necessity to initiate and coordinate cooperation between appropriate institutions of support. Probation officers must face bureaucracy, which often hampers and delays effective provision of help to their clients. The conflict-prone character of this occupation is also expressed in its need to combine two contrasting demands: on the one hand, those of a formal agent executing court rulings, and on the other, those of an informal helper seeking to improve the social, legal, economic, and psychological conditions of their clients [3].

The work of a probation officer is highly stressful [4] and occupational burnout is frequent [5]. The current study focused on this issue and analyzed it with respect to styles of coping with stress. Its aim was thus to analyze the relationship between coping styles and the likelihood of experiencing occupational burnout symptoms among probation officers. Our research is important because it addresses the important problem of the relationship between coping styles and professional burnout. Research [6] shows that overworked and stressed employees are more exposed to burnout and their individual reactions to stress cannot be ignored in social and psychological studies concerning this matter. Working of this problem required analysis of socio-demographical variables such as age, sex, work experience, and specialization, which were identified by White et al. [7], Pitts [8], and Simmons et al. [9] as factors related to burnout.

The study was established by way of statistical analysis of data from two research methods: the Coping Inventory for Stressful Situations (CISS) and the Maslach Burnout Inventory (MBI). The analyses also included such factors as work experience, probation specialization, gender, and age as potential determinants of occupational burnout.

Workplace stress is a phenomenon that has been well recognized. Many psychological and social studies provided a lot of knowledge about the complexity of this kind of stress, its factors and intensity. However, the specificity of workplace stress depends on the type of professional groups that are exposed to it. Therefore, the diagnosis of particular workplace stress determinants and its psychological predictors is important. This article is an attempt of diagnosis that is grounded in the Polish context of working of probation officers. The justification for undertaking such research was the fact that earlier this professional group was not covered by them (to such a wide extent), and due to the significant differences between the system of probation in Poland and such systems in other countries, the use of other studies to describing this phenomenon was insufficient and could lead to erroneous conclusions. Piotrowski’s [10] research on correctional staff verified the role of styles of coping with stress in explaining the overall level of burnout in multiple regression. The author did not undertake the analysis of the multiple regression model for individual dimensions of burnout (emotional exhaustion, a sense of personal achievements, and depersonalization) and styles of coping with stress. An important area of Piotrowski’s research was to examine the differences between individual personality dimensions using the Costa and McCrae’s NEO-PI-R test and the overall result of occupational burnout. We plan to investigate the relationship between probation officer burnout and personality in further research.

The results of this study are important because of the practical applications. Firstly, probation officers themselves can use them, because thanks to them it is possible to learn the best strategies for coping with stress and professional burnout. Secondly, our study may also encourage employers of probation officers to take measures to protect employees from severe stress, as its consequence is lower efficiency of their work.

### The Nature of Probation Officers’ Work in Poland

The Polish court probation differs from similar institutions functioning in other countries. Founded in 1919, it has a long tradition [11,12]. However, its beginnings were different than in those countries where the institution of probation was created: the USA and the UK [13,14,15]. Compared with those countries, in Poland, juvenile probation was created first, and probation for convicts was created much later. The direction of development of Polish court probation was also influenced by Communist rule. It meant a specific isolation and a lack of free access to methods of work and organization, which functioned in central European countries. In Poland, a model of court probation has developed, which functions organizationally within the structure of the courts. Probation officers work in probation teams, managed by directors. The teams work in district courts, whereas the regional probation officer works in the regional court. The regional probation officer is subordinated to the president of the regional court and manages probations in its jurisdiction.

Two specializations exist in the Polish court probation system: adult probation—responsible for executing criminal court rulings—and family and juvenile probation—responsible for executing the rulings and activities ordered by family and juvenile courts. Probation officers work in only one specialization, but they can change them at will. The consequence of this system is a high variability of cases in which probation officers are involved. The Polish system differs in this respect from similar systems in other countries. Adult probation officers are responsible for such tasks as supervising convicts; carrying out community sentences; supervising convicts in carrying out court-ordered responsibilities (if not supervised by administrators); carrying out electronic supervision; preparing convicts for release; supporting convicts leaving penitentiary institutions; carrying out community interviews, and carrying out select detention orders, for example, restraining orders. On the other hand, family and juvenile probation officers are responsible for supervising the carrying out of parental responsibility, supervising minors and individuals in withdrawal treatment; supervising minors and their families or legal guardians in carrying out court-ordered responsibilities; taking individuals out of parental custody or care; participating in the parents’ contact with the child when ordered by the court, and carrying out community interviews. Probation officers of both specializations also participate in interdisciplinary teams working to prevent domestic violence on the municipal level. As the above suggests, probation officers in Poland carry out numerous tasks. In other countries, some of these tasks are carried out by other services. For example, social workers often supervise the carrying out of parental responsibility.

A notable aspect of the Polish court probation system is the requirement to work with the clients in their place of residence. Working in the clients’ communities has many benefits for the probation officers. They can gain more knowledge about their clients. They can also utilize support from the clients’ significant others, such as spouses, parents, or legal guardians. By influencing the clients’ environment, the probation officers also indirectly influence the clients themselves. Such a strategy (working with clients in their environment) often yields very good results [16,17]. However, it can also cause various risks: aggression from the clients or others, aggression from house pets (dogs), or difficulties working with individuals suffering from mental disorders or addictions.

Higher education in pedagogy, psychology, sociology, or law is required to become a probation officer in Poland. The majority (64%) have education in pedagogy. Additionally, almost 80% are women. The mean age of Polish probation officers is 49 years. The average work experience of probation officers is 16 years, with general work experience being 22 years. For 21%, being a probation officer is their first job [18]. There are around 5200 professional probation officers in Poland, including around 2000 family and juvenile probation officers. They are supported by social probation officers who work as volunteers. Probation officers can be promoted to senior and specialist probation officers [19].

The court probation system is highly susceptible to changes in criminal policy. The phenomenon of crime is increasingly frequently politicized, with crime prevention methods becoming part of various parties’ election programs [20,21]. This causes frequent changes in law and in the organization of the court probation system. The latter were at times very radical, for example, in England, Wales [22,23,24], or France [25]. The Polish system has also been affected by these processes. They have largely concerned numerous legal changes.

The psychosocial consequences of stress in the work of probation officers have been identified as a significant problem area in scientific research for a long time. Existing studies confirm that probation officers experience role conflicts, concerns over personal safety, a lack of administrative and supervisory support, high job rotation, a lack of participation in organizational decision making, low pay, limited opportunities for career advancement, and contend with excessive bureaucratic demands, overly lenient court rulings, and a lack of appreciation for their work by the public [26,27,28,29,30,31,32]. The most significant consequence of occupational stress is occupational burnout. Maslach defines occupational burnout as “a psychological syndrome, a prolonged reaction to chronic, stressful situations which take place between people at work” [33] (p. 56). She also notes that long-term exposure to various social stressors and their consequences leads to the process of psychological erosion, which makes evident the psychological and social consequences of stress in addition to the physical. Schaufeli and Peeters [34] (p. 20) identified burnout as a “long-term stress reaction that occurs among professionals who … do people work”. Burnout among probation officers can involve the aspects of emotional exhaustion, depersonalization, and a reduced sense of personal accomplishment [35]. The negative effects of burnout cause absences from work, exhaustion [36], and an attitude of cynicism [35]. The main predictors of burnout among probation officers are a low level of institutional (workplace) responsibility and lack of ability to diagnose and manage organizational problems [5], which may cause employees to feel a reduced sense of agency over the institution’s functioning, lack of autonomy, lack of clarity in the understanding of the institution’s aims and rules; excessive workloads, perceived lack of support and motivation from supervisors [37]; lack of precise definitions of the roles and functions in the institution, which leads to conflicts [38], as well as lack of appropriate communication between workers and supervisors [39].

The issue of occupational burnout is studied in the context of many occupations, although it remains underexplored in the specific context of probation officers [40,41]. The presence of burnout in this group is not surprising, seeing the important role of probation officers in criminal policy and in facilitating public safety and rehabilitation of convicts [8]. Moreover, the specifics of the clients’ serious criminal offences, as well as the probation officers’ experiences of verbal and physical assaults in contact with these clients are related to higher burnout levels [41]. Another significant variable is the client’s environment in which probation officers carry out their work and its relation with burnout and professional competence [7].

Dewe showed a relationship between subjectively experienced stress and capabilities and strategies of coping with workplace demands [42]. The choice of an appropriate coping style is important for increasing or decreasing burnout symptoms [43]. Coping styles can have a positive or a negative character. The former includes seeking support from colleagues and significant others as well as stress coping workshops and trainings. Negative coping styles include withdrawal, excessive self-criticism, and substance abuse [44]. Existing research [45,46] shows that coping skills are a significant protective factor against burnout. Researchers of burnout [47,48,49,50] claim that problem-focused coping results in lower burnout. There are also links between results on individual burnout scales and the choice of an appropriate coping style.

## 2. Materials and Methods

### 2.1. Aim of the Study

The main aim of the study was to assess the relationship between occupational burnout levels among Polish probation officers and styles of coping with stress that they prefer. Based on the theoretical considerations presented in the introductory paragraph, the following research questions were formulated:

1. Does coping style influence the emergence of occupational burnout symptoms?
2. Is work experience as a probation officer related to occupational burnout levels?
3. Is the probation officers’ specialization related to occupational burnout levels?
4. Is the probation officers’ gender related to occupational burnout levels?
5. Is the probation officers’ age related to occupational burnout levels?

### 2.2. Procedure

The study used psychological measures. It was carried out in nine randomly selected court districts in Poland where probation offices are located. A total of 1000 probation officers received the questionnaire set, out of which 602 have returned them. Two hundred and twelve sets were excluded from the analysis due to various formal issues, for example missing data or patterns strongly suggesting random answers. Three hundred and ninety complete questionnaire sets were included in the data analysis. It was carried out using the IBM SPSS Statistics 26.0 software. The multiple regression model, parametric significance test for independent samples, and A. Hayes’ PROCESS macro for moderation analyses were used.

It is important to highlight that all participants gave their informed consent for inclusion before they participated in the study. They were informed about the anonymity, the data protection procedure and the guarantee of privacy.

### 2.3. Participants

The research sample consisted of 390 professional probation officers. The majority were women, which reflects the distribution of the population of this professional group in Poland. The largest group of probation officers are people aged 38 to 48. Every fifth respondent is in the 49–64 age group. The average age of a probation officer is 40 years (*M* = 40.72, *SD* = 8.60). In most cases, participants had less than 14 years of professional experience. The most experienced constitute 3% of research participants. The average work experience in the study group is 10 years (*M* = 10.51, *SD* = 7.27). More than half of the respondents deal with the enforcement of court decisions in criminal cases, while slightly fewer work with the family and minors.

Table 1 shows a detailed description of the sample, including frequencies and percentages.

### 2.4. Measures and Strategy of Analysis

The study utilized the Coping Inventory for Stressful Situations (CISS) by N. S. Endler and J. D. A. Parker in the Polish adaptation by J. Strelau, K. Wrześniewski, and P. Szczepaniak [51]. The CISS comprises 48 items describing various behaviors in stressful situations. The respondents indicate, on a five-point scale, the likelihood of engaging in a given behavior in a difficult, stressful situation. The results are described on three scales:

1. PFS—problem-focused style. Individuals scoring high on this scale attempt to solve problems in stressful situations by cognitive reframing or trying to change the situation.
2. EFS—emotion-focused style, characteristic for individuals who display a tendency to focus on the self and own emotions (anger, guilt, tension) in stressful situations. They are also characterized by wishful thinking and fantasizing. On the one hand, these behaviors may decrease emotional tension, though, on the other hand, they may increase it.
3. AFS—avoidance-focused style, characteristic for individuals who avoid thinking about or engaging with stressful situations. The AFS can assume two forms: TAO—task-oriented avoidance, that is, seeking distraction, and POA—person-oriented avoidance, that is, social diversion.

The second measure used was the Maslach Burnout Inventory (MBI) created by Maslach and Jackson. It allows for assessing three aspects of the occupational burnout syndrome: emotional exhaustion, depersonalization, and a sense of personal accomplishment. It includes 22 items divided into three unequal groups, with each group concerning one aspect. The emotional exhaustion scale comprises nine items, the depersonalization scale of five items, and the sense of personal accomplishment scale of eight items. The items in the emotional exhaustion and depersonalization are formulated negatively, while the items in the sense of personal accomplishment are formulated positively. Thus, results are calculated separately for each scale. High scores on the emotional exhaustion and depersonalization, and low levels of personal accomplishment are associated with high levels of burnout. The MBI has good psychometric properties and is the most frequently used measure in studies of burnout [52,53]. It is multidimensional in nature and was thus used to examine whether the three dimensions of burnout are related to different coping styles among Polish probation officers. A demographic survey was also included with the two measures.

## 3. Results

In order to answer whether, and to what extent, coping styles allow for predicting the emergence of burnout symptoms among probation officers, a multiple regression analysis was carried out. The explained variable were the three dimensions of burnout: emotional exhaustion, depersonalization, and personal accomplishment. The explanatory variables were the coping styles: problem-focused, emotion-focused, and avoidance-focused, divided into task-oriented and person-oriented avoidance.

The Cronbach’s alpha obtained in the sample for the measures used in the study allows to infer appropriate reliability (Table 2).

Table 3 shows multiple regression analysis results for three dimensions of burnout and coping styles among probation officers.

Emotional exhaustion is characterized by a feeling of overwork and depletion of emotional and physical resources due to excessive workplace demands. The analysis of the assumed regression model shows that styles of coping in stress explain 23% of the emotional exhaustion levels among probation officers.

The probation officers’ coping styles explained 18% of personal accomplishment. Personal accomplishment is defined as a measure of feelings of competence and successful achievements in one’s work.

Depersonalization refers to the interpersonal aspect and is characterized by a negative, unengaged, and overly distanced approach to task realization and other aspects of work. In time, a depersonalized individual becomes withdrawn and limits their workplace effectiveness both quantitatively and qualitatively. This leads to such negative consequences as dehumanization, loss of ideals and values, and negative interactions with colleagues. According to multiple regression analysis, styles of coping in stress explain 12% of the depersonalization level among probation officers.

The multiple regression models for coping styles in stress and respective dimensions of burnout are presented in Table 4, Table 5 and Table 6.

From among the coping styles included in the analysis, the emotion-focused style had a statistically significant impact on the emergence of emotional exhaustion in the participants (beta = 0.48, *p* < 0.05). The remaining coping styles included in the multiple regression model did not have a statistically significant association with emotional exhaustion.

Table 5 shows that problem-focused coping was related the most strongly to the explained variable (beta = 0.27, *p* < 0.05). This means that among probation officers, a high sense of personal accomplishment is accompanied by engaging in behaviors aimed at solving problems or changing the stressful situation using cognitive processes. The emotion-focused coping style correlated negatively and proportionally with the sense of personal accomplishment (beta = −0.26, *p* < 0.05), meaning that the higher the probation officers’ scores on the emotion-focused coping scale, the lower their scores on the sense of personal accomplishment scale. The lower scores on the sense of personal accomplishment manifests in a reduced sense of own competences and work effectiveness. It can be related to depressive tendencies and difficulties in coping with workplace demands. Such a state can be exacerbated by a lack of or inadequate support from the environment. It can also be related to feelings of pointlessness and resignation. Such probation officers can perceive themselves to be lacking the necessary helping skills, which can lead to a harmful belief about own uselessness [54]. The third variable in the assumed regression model which correlated with the sense of personal accomplishment among probation officers was the coping style of person-oriented avoidance (beta = 0.24, *p* < 0.05). This means that probation officers experiencing a reduced sense of personal accomplishment avoid thinking about important problems and avoid attempts at solving stressful situations. They attempt to escape from the problems by engaging in distractions—they might, for example, go shopping, clean their house, sleep, watch TV, or seek interpersonal contacts.

In Table 6, the analysis of the assumed regression model shows that styles of coping in stress explain 16% of the depersonalization levels among probation officers. This relationship is statistically significant. In the assumed multiple regression model, the three of four coping styles were related (statistically significant) to the explained variable of depersonalization: beta = 0.16, *p* < 0.05 for the emotion-focused style, beta = 0.12, *p* < 0.05 for the task-oriented avoidance style. The relationship between depersonalization and person-oriented avoidance was inversely proportional, beta = −0.14, *p* < 0.05.

Moreover, to answer the question of whether such variables as work experience and probation specialization, gender, or age significantly differentiate probation officers with respect to occupational burnout levels, a statistical analysis using Fisher’s nonparametric tests and Student’s *t* tests was carried out (Table 7).

The reduced sense of personal accomplishment most strongly differentiated the groups with the most and the least work experience. Depersonalization also differentiated the groups, but it was lower in the most experienced group. Depersonalization is exhibited through a negative, unengaged, and overly distanced approach to task realization and other aspects of work. In time, a depersonalized individual becomes withdrawn and limits their workplace effectiveness both quantitatively and qualitatively. This leads to such negative consequences as dehumanization, loss of ideals and values, and negative interactions with colleagues. Emotional exhaustion differentiated the most strongly between the group in the mid-point of their career and the least experienced group. Emotional exhaustion is characterized by a sense of overwork, depletion of emotional and physical resources, and excessive exploitation. It develops the most intensely during at the initial and advanced stages of employment, which is confirmed in the research. In their study of a sample of probation officers, Gayman and Bradley [40] concluded that work experience is positively associated with occupational burnout and have estimated that this association increases each year. Younger employees, especially those with less work experience, exhibit higher burnout scores than older and/or more experienced employees [55]. Total burnout score also significantly differentiated the groups of probation officers.

An analysis of the data in Table 8 confirms the statistically significant association between emotional exhaustion and total burnout scores on the one hand, and the probation specialization on the other. The data show that family and juvenile probation officers are more prone to the negative consequences of workplace stress than adult probation officers.

The obtained results lead to the conclusion that the probation officers’ gender influences the emotional exhaustion dimension of occupational burnout to a statistically significant degree. Female probation officers experience emotional exhaustion significantly more often than male probation officers (Table 9).

A reduced sense of professional accomplishment was characteristic for the youngest (23–37 years old) and the oldest (49–64 years old) groups (Table 10). Probation officers in the 38–48 age group scored higher on depersonalization, which suggests an ongoing process of the dehumanization of the work they perform. This group was also characterized by the highest levels of emotional exhaustion. However, it must be noted that this is also the period of the most intense professional activity. Similar effects were observed for the total burnout score.

In order to test the moderating role of sociodemographic factors (gender, work experience, age, and probation specialization) in the relationship between coping styles and occupational burnout, a moderation analysis using A. Hayes’ PROCESS macro [56] was carried out. The analysis showed that age, work experience, and probation specialization were not statistically significant moderators of the relationship between the CISS and MBI scores. Gender was a statistically significant moderator only in four of the models. These models are described in greater detail below.

Model 1. The moderating role of gender in the relationship between problem-focused coping style (PFS) and emotional exhaustion.

The model was revealed to have a good fit to data, *F*(3.385) = 3.65; *p* = 0.013, and it explained around 3% of the variance in emotional exhaustion (R^2^ = 0.028). Table 11 shows the model’s regression coefficient values.

**Figure 1:**
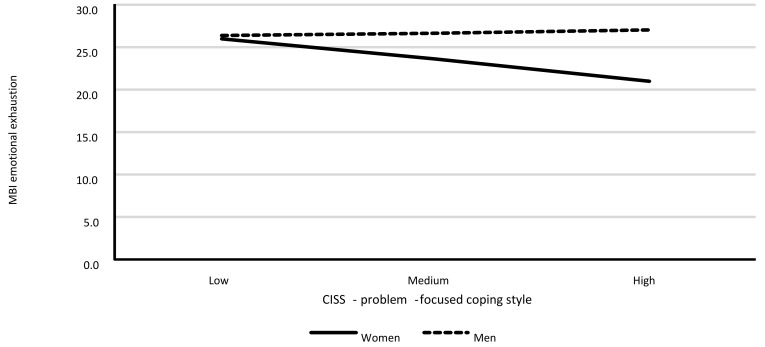
Associations between problem-focused coping (PFS) and emotional exhaustion for men and women separately.

The analysis showed a lack of statistically significant associations between PFS and emotional exhaustion, a statistically significant relationship between gender and emotional exhaustion, as well as a statistically significant interaction effect of PFS and gender on emotional exhaustion. A detailed analysis showed a statistically significant relationship between PFS and emotional exhaustion only for women (*B* = −0.33; *SE* = 0.14; *p* = 0.018). As PFS scores increased in women, the level of emotional exhaustion decreased. This association was not statistically significant for men (*B* = 0.04; *SE* = 0.09; *p* = 0.598). Results are shown in Figure 1.

Model 2. The moderating role of gender for the relationship between the person-oriented avoidance type of the avoidance-focused coping style (POA) and emotional exhaustion.

This model also had a good fit to data, *F*(3.385) = 4.72; *p* = 0.003, and it explained 3.6% of the variance in emotional exhaustion (R^2^ = 0.036). Table 12 shows the model’s regression coefficient values.

**Figure 2:**
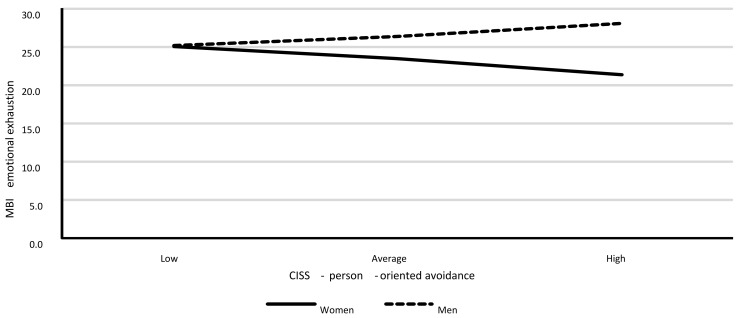
Associations between POA and emotional exhaustion for each gender separately.

The analysis showed a lack of statistically significant associations between POA and emotional exhaustion, a statistically significant association between gender and emotional exhaustion, as well as a statistically significant interaction effect of gender and POA on emotional exhaustion. The analysis of interaction effects showed a statistically significant association between POA and emotional exhaustion for men (*B* = 0.42; *SE* = 0.17; *p* = 0.015). As POA scores increased in men, emotional exhaustion also increased. For women, this association was significant only at the level of a statistical trend (*B* = −0.52; *SE* = 0.30; *p* = 0.079), with this association taking a negative direction. The results are shown on Figure 2.

Model 3. The moderating role for the relationship between the problem-focused coping style (PFS) and depersonalization

Model 3 also had a good fit to data, *F*(3.385) = 3.50; *p* = 0.016, and it explained around 3% of the variance in depersonalization (R^2^ = 0.027). Table 13 shows the model’s regression coefficient values.

**Figure 3:**
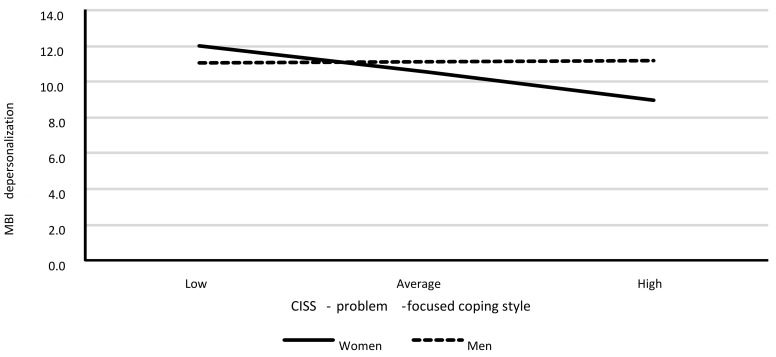
Associations between PFS and depersonalization, separately for both genders.

The analysis showed a lack of statistically significant associations between PFS and depersonalization, as well as a lack of statistically significant associations between gender and depersonalization. However, the interaction of both these variables was statistically significant. A detailed analysis showed a statistically significant association between PFS and depersonalization only for women (*B* = −0.20; *SE* = 0.06; *p* = 0.002). As the PFS scores increased in women, the level of depersonalization increased. For men, this association was not statistically significant (*B* = 0.01; *SE* = 0.04; *p* = 0.821). The results are shown in Figure 3.

Model 4. The moderating role for the association between person-oriented avoidance (POA) and depersonalization.

The last of the analyzed models was revealed to have an insufficient fit to data, *F*(3.385) = 1.74; *p* = 0.159 (the model explained less of the variable’s variance than its mean), and it explained around 1% of the variance in emotional exhaustion (R^2^ = 0.013). This model has to be interpreted with care. Table 14 shows the model’s regression coefficient values.

**Figure 4:**
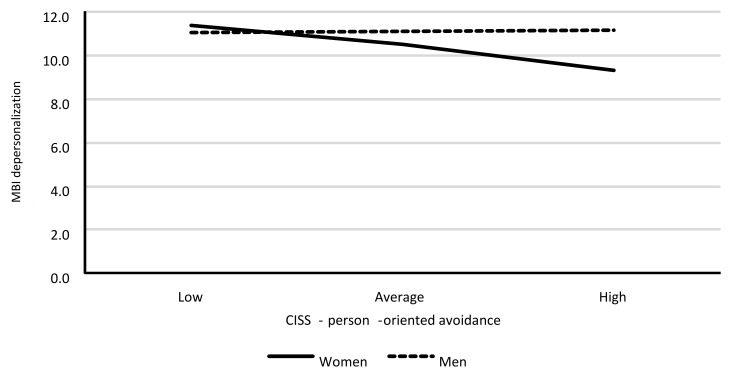
Associations between POA and depersonalization, separately for each gender.

The analysis showed a lack of statistically significant associations between POA, gender, and depersonalization. However, the interaction of both of these variables was statistically significant. The interaction between POA and depersonalization was statistically significant only for women (*B* = −0.30; *SE* = 0.14; *p* = 0.029). As the POA scores increased in women, depersonalization scores decreased. This association was not statistically significant for men (*B* = 0.02; *SE* = 0.08; *p* = 0.190). Results are shown in Figure 4.

## 4. Discussion

The examination of the relationship between coping styles with stress and the likelihood of experiencing occupational burnout symptoms among probation officers in Poland was the aim of this study. The analysis of the empirical data allows us to draw conclusions about the significant importance of styles of coping with stress in explaining the phenomenon of occupational burnout among probation officers. The role of coping styles in explaining individual burnout syndromes has been identified. It can be assumed that coping with stress by using an emotion-focused style is conducive to the burnout of probation officers. In order to test the moderating role of sociodemographic variables for the relationship between coping strategies and occupational burnout, a number of moderation analyzes were carried out, which indicated that in the case of as many as four models presented, gender as the one of the sociodemographic and organizational variables played an important role as a moderator. In the effective prevention of burnout, both personality traits, psychological skills, and organizational conditions are important. The condition for effective prevention of occupational burnout is the use of proven forms, e.g., anti-stress workshops, which should be held regularly and should be particularly targeted at risk groups. Probation officers as a professional group have been often overlooked in research on occupational burnout. Therefore, our study leads not only to theoretical but also to practical implications and the matter is significant for the functioning of probation officers service in Poland.

Helping professions involve very high stress levels, since social service workers must be proficient in managing a wide variety of problems. They participate in conflict-prone relationships with their clients and are constantly faced with the specific demands of their clients’ environments. They also participate in competence conflicts between the institutions they cooperate with. Noworol and Marek’s [57] occupational burnout model posits the existence of specific micro-paths of burnout for each profession. The authors point out that not only the work environment, together with its specific demands, but also work which is incongruent with personal psychosocial competences or professional identity are predictors of burnout. Such conditions can quickly lead to the emergence of the burnout process. This approach fits the specifics of probation officer work. Maslach and Leiter [58] state that the MBI focuses on individuals’ personal experiences with work, and they highlight the strong relationship between burnout and work. They also underscore the fact that burnout develops very quickly in the current professional climate. The chief reason behind this is the significant gap between work demands and the worker’s characteristics as well as the social context, in which common human values are less important than economic effects. Researchers report that burnout is often perceived as an individual issue, and they strongly advise against such a perspective. Instead, they hold that burnout is an issue of the social environment in which a given person works. The current study confirms this, as the emotion-focused coping style was found to facilitate burnout among probation officers. The coping style focused on own emotional experiences such as anger, guilt, and tension is characteristic for individuals who prefer wishful thinking and fantasizing rather than effective and rational behaviors aimed at removing or minimizing the stressor. Behaviors associated with this coping style are chiefly aimed at lowering the emotional tension accompanying the stressful situation. Not undertaking concrete, problem-solving action often causes directly opposite results, further increasing psychological tension and negative emotions [59]. Among the respondents in the current study, a sense of high personal accomplishment was accompanied by problem-solving activities or changing the stressful situation using cognitive processes. Probation officers experiencing a reduced sense of personal accomplishment tended to avoid thinking about important problems. This is characteristic for the avoidance-focused coping style, which also includes avoidance of experiencing and problem-solving in the stressful situation. Individuals using this coping style distract themselves from their problems—they might, for example, go shopping, clean their house, sleep, watch TV, or seek interpersonal contacts. White et al. [60] studied coping styles among adult probation officers and identified many adaptive mechanisms related to a healthy lifestyle. Most of them were related to the ability to identify and adequately respond to negative situations and emotions. These included a sense of humor, maintaining positive family relationships, having interests, maintaining stable social relationships, taking part in trainings, physical activity, goal setting, and being an authority in professional matters.

The current study showed that probation officers with the shortest (1–14 years) and longest (26–40) work experience differ with respect to the burnout aspect of depersonalization. Emotional exhaustion was also characteristic for the probation officers with an average amount of work experience (15–25 years). Probation officers in Poland have limited opportunities for career advancement—only three promotion ranks, with the highest one being achievable within the first ten years of work, if conditions are favorable. This can cause significant limitations in mobilizing individual potential, seeking innovative methods of work and personal involvement in work. Moreover, heightened stress levels can result in high job rotation, which is the most frequently cited reason for quitting [28]. This association was also confirmed by Lewis et al. among probation officers [41]. The largest group of probation officers who quit their work, according to Lee et al., were between 20 and 34 years old and had low work experience (up to 3 years) [61].

The current study shows that family and juvenile probation officers are at greater risk for negative consequences of occupational stress, according to the total burnout and emotional exhaustion scores. This can be a result of the specific demands of their work, insufficient regulations in the Polish law, or a lack of a systemic solution in the form of peer supervision.

Probation officers in Poland are predominantly women, who experience emotional exhaustion more often compared to men. This is an important organizational problem, as legal regulations of the profession of probation officer do not consider this difference, nor other commonly established knowledge about the demands placed on women in various social roles (e.g., as mothers). The results of the current study confirm those by Gayman and Bradley [40] who showed that female probation officers exhibited higher burnout levels than did male probation officers. Wells, Colbert, and Slate [29] verified the association between gender and stress in a sample of probation officers. They found that women experienced higher physical stress and were more likely to use sick leave, whereas men, despite a subjectively high experience of stress, continued working, putting themselves at risk of significant health consequences. The results of Wells et al. can testify to the consequences of assuming other social roles in addition to the professional one, as indicated above. Men can avoid disclosing mental health problems out of fear of having their competence judged negatively.

The current study shows that for female probation officers, the problem-focused coping style leads to lower levels of emotional exhaustion and depersonalization. For women, this association also occurred between the avoidance-focused style (person-oriented avoidance) and lower depersonalization levels. For men, engaging in this coping style leads to higher emotional exhaustion. However, for women, a tendency towards lower emotional exhaustion was observed.

Probation officers in the age range of 38–48 years achieved higher depersonalization scores and were also characterized by the highest emotional exhaustion levels. This association can be explained by the different challenges faced in the social sphere, as well as the nature of Polish probation officers’ work, who are not able to retire earlier in contrast to other, related services, such as the police or the prison service.

## 5. Conclusions

In sum, a statistically significant relationship between preferred styles of coping with stress and the occupational burnout syndrome exists for probation officers. The emotion-focused coping style facilitates emotional exhaustion among probation officers. Thus, effective and rational coping aimed at removing or minimizing stressors should be promoted instead of wishful thinking and fantasizing. Because of its character, the work of a probation officer involves many stressful situations. Thus, there exists a real need for probation officers to engage in behaviors oriented at reducing the accompanying emotional tension. Not undertaking concrete, problem-solving action often causes directly opposite results, further increasing psychological tension and negative emotions. Using cognitive processes intended to solve problems or change the stressful situation facilitates a sense of high professional achievement among probation officers, which protects them against a sense of incompetence, low productivity, and critical self-esteem.

A necessary element of effective burnout prevention involves changes in workplace organization, taking into account the employee’s relationship with their work and considering the emerging problems not only from the employee’s perspective, but also that of the workplace. The main goal of the organizational strategy should be to create structural and management procedures aimed at promoting engagement and preventing burnout. To this end, data on the organization pertaining to six areas of interest (workload, control, pay, community, justice, and values) as well employee engagement should be gathered. Next, appropriate interventions should address the structure and practices of the management [58]. Management solutions that promote innovation, increasing employee competences, motivating and rewarding engaged probation officers, as well as improving communication between judges, probation officers, and management should be considered [62] (p. 92).

## 6. Strengths, Limitations, and Future Research

One strength of the current study was its large sample, which included probation officers from all over Poland, specializing both in adult as well as in family and juvenile cases. This allowed for capturing the specifics of coping styles used by probation officers as well as the associations between these styles and burnout levels. However, the current study also has some limitations. One is the fact that it involved only probation officers in Poland. This has already been addressed in an ongoing research project seeking to include probation officers from other countries. Such a project is possible because the employed measures, the Coping Inventory for Stressful Situations (CISS) and the Maslach Burnout Inventory (MBI), are standardized. Another limitation is the lack of in-depth interviews with the probation officers. Such data would allow for a more detailed description of situations that probation officers find especially stressful, as well as the coping styles they use in specific circumstances. This is also addressed by the research project in development. Thus far, studies involving juvenile probation officers showed that they exhibited limited strategies of coping with burnout symptoms. They have also reported that burnout had a negative influence on their relationships with their clients (they became less tolerant or more demanding). A few probation officers indicated that support from coworkers and supervisors helped them cope with the feeling of burnout. Others considered support from the family to be the most important [63]. Of interest is also the result that the quality of the relationship with socially maladjusted youth determined the probation officers’ task effectiveness [64]. This suggests that further studies of probation officers using qualitative and quantitative measures are warranted.

## Figures and Tables

**Table 1:** Demographic characteristics of the sample.

Variables		N	%
Gender	Women	289	74%
	Men	101	26%
Age	23–37	145	37%
	38–48	168	43%
	49–64	77	20%
Specialization	Criminal cases	219	56%
	Family and juvenile cases	171	44%
Work experience	1–14	259	67%
	15–25	119	30%
	26–40	12	3%

**Table 2:** The Cronbach’s alpha index.

Variable	Cronbach’s Alpha
Emotional exhaustion	0.812
Personal accomplishment	0.789
Depersonalization	0.813
Problem-focused style	0.798
Emotion-focused style	0.788
Person-oriented avoidance style	0.821
Task-oriented avoidance style	0.841

**Table 3:** Multiple regression analysis results for emotional exhaustion, personal accomplishment, depersonalization, and coping styles.

Dependend Variable	Adjusted R2	MS Model	Residual df	Residual MS	F	*p*
Emotional exhaustion	0.23	10,365.30	390	89.08	116.34	0.000
Personal accomplishment	0.18	1602.02	390	54.06	30.28	0.050
Depersonalization	0.12	414.52	390	21.31	10.26	0.000

**Table 4:** Multiple regression model, the three coping styles as predictors to the explained variable of emotional exhaustion.

Variable	Emotional Exhaustion Beta	*p*
Problem-focused style	0.07	0.131
Emotion-focused style	0.48	0.000
Person-oriented avoidance style	−0.00	0.899
Task-oriented avoidance style	0.07	0.121

**Table 5:** Multiple regression model, the three coping styles as predictors to the explained variable of the sense of personal accomplishment.

Variable	Sense of Personal Accomplishment Beta	*p*
Problem-focused style	0.27	0.000
Emotion-focused style	−0.26	0.000
Person-oriented avoidance style	0.24	0.042
Task-oriented avoidance style	0.05	0.246

**Table 6:** Multiple regression model, the three coping styles as predictors to the explained variable of depersonalization.

Variable	Depersonalization Beta	*p*
Problem-focused style	0.03	0.445
Emotion-focused style	0.16	0.000
Person-oriented avoidance style	-0.14	0.024
Task-oriented avoidance style	0.12	0.020

**Table 7:** Intergroup means comparison for work experience and burnout (total score and individual scales).

MBI Scales	1–14		15–25		26–40		F	*p*
	M	SD	M	SD	M	SD		
Personal accomplishment	37.58	8.00	35.43	8.11	38.18	11.02	2.88	0.035
Depersonalization	11.11	5.21	11.38	4.35	8.64	3.13	4.09	0.007
Emotional exhaustion	24.98	10.05	29.60	11.56	22.00	9.45	9.30	0.000
Total score	24.55	4.77	22.93	4.25	24.78	4.92	3.82	0.010

**Table 8:** Intergroup means comparison for specialization type and burnout (total score and individual scales).

MBI Scales	Adult Probation Officers		Family and Juvenile Probation Officers		t	*p*
	M	SD	M	SD		
Personal accomplishment	36.58	8.17	37.24	7.92	0.020	ns.
Depersonalization	10.91	5.04	11.30	4.79	0.010	ns.
Emotional exhaustion	24.94	10.17	27.47	11.32	3.31	0.022
Total score	24.14	4.73	25.33	5.10	1.26	0.018

**Table 9:** Intergender means comparison for burnout (total score and individual subscales).

MBI Scales	Women		Men		t	*p*
	M	SD	M	SD		
Personal accomplishment	36.80	8.31	37.53	7.56	1.62	ns.
Depersonalization	11.11	4.92	10.76	4.87	0.429	ns.
Emotional exhaustion	26.74	11.06	23.98	9.53	5.11	0.018
Total score	24.88	5.12	24.09	4.27	3.87	ns.

**Table 10:** Intergroup means comparison for age and burnout (general score and individual scales).

MBI Scales	23–37		38–48		49–64		F	*p*
	M	SD	M	SD	M	SD		
Personal accomplishment	37.39	8.43	36.07	7.50	38.12	8.72	1.99	ns.
Depersonalization	11.02	5.06	11.80	4.90	9.52	4.39	5.79	0.003
Emotional exhaustion	24.23	9.68	28.62	10.54	24.14	12.04	8.33	0.000
Total score	24.21	4.65	25.49	4.91	23.92	5.32	3.86	0.022

**Table 11:** Unstandardized regression coefficients for Model 1.

			95% CI	
	B	SE	LL	UL
Constant	25.93 ***	0.54	24.86	26.99
PFS	−0.05	0.07	−0.19	0.09
Gender	3.10 *	1.24	0.66	5.55
PFS × Gender	0.38 *	0.16	0.06	0.70

* *p* < 0.050; *** *p* = 0.000; SE: Statistical indexes.

**Table 12:** Unstandardized regression coefficients for Model 2.

			95% CI	
	B	SE	LL	UL
Constant	25.75 ***	0.54	24.67	26.82
POA	0.18	0.15	−0.11	0.47
Gender	3.20 *	1.28	0.69	5.72
POA × Gender	0.94 **	0.34	0.27	1.62

* *p* < 0.050, ** 0.050 > *p* > 0.005; *** *p* = 0.000.

**Table 13:** Unstandardized regression coefficients for Model 3.

			95% CI	
	B	SE	LL	UL
Constant	10.96 ***	0.25	10.48	11.45
PFS	−0.05	0.03	−0.11	0.02
Gender	0.57	0.57	−0.55	1.69
PFS × Gender	0.21 **	0.07	0.06	0.36

** *p* < 0.005; *** *p* = 0.000.

**Table 14:** Unstandardized coefficients for Model 4.

			95% CI	
	B	SE	LL	UL
Constant	25.93 ***	0.54	24.86	26.99
POA	−0.05	0.07	−0.19	0.09
Gender	3.10 *	1.24	0.66	5.55
POA × Gender	0.38 *	0.16	0.06	0.70

* *p* < 0.050; *** *p* = 0.000.

## Data Availability

Data available on request due to restrictions e.g., privacy or ethical. The data presented in this study are available on request from the corresponding author. The data are not publicly available due to the privacy and professional specificity of the people who took part in this research and who work the Polish probation system.
